# Big genomics and clinical data analytics strategies for precision cancer prognosis

**DOI:** 10.1038/srep36493

**Published:** 2016-11-07

**Authors:** Ghim Siong Ow, Vladimir A. Kuznetsov

**Affiliations:** 1 Bioinformatics Institute, 30 Biopolis Street #07-01 Matrix, 138671 Singapore; 2 School of Computer Engineering, Nanyang Technological University, 639798 Singapore

## Abstract

The field of personalized and precise medicine in the era of big data analytics is growing rapidly. Previously, we proposed our model of patient classification termed Prognostic Signature Vector Matching (PSVM) and identified a 37 variable signature comprising 36 let-7b associated prognostic significant mRNAs and the age risk factor that stratified large high-grade serous ovarian cancer patient cohorts into three survival-significant risk groups. Here, we investigated the predictive performance of PSVM via optimization of the prognostic variable weights, which represent the relative importance of one prognostic variable over the others. In addition, we compared several multivariate prognostic models based on PSVM with classical machine learning techniques such as K-nearest-neighbor, support vector machine, random forest, neural networks and logistic regression. Our results revealed that negative log-rank p-values provides more robust weight values as opposed to the use of other quantities such as hazard ratios, fold change, or a combination of those factors. PSVM, together with the classical machine learning classifiers were combined in an ensemble (multi-test) voting system, which collectively provides a more precise and reproducible patient stratification. The use of the multi-test system approach, rather than the search for the ideal classification/prediction method, might help to address limitations of the individual classification algorithm in specific situation.

In the era of big data and with the development of national or international electronic healthcare records, large and comprehensive databases of genomic, transcriptomics, proteomics or metabolomics variables, as well as traditional clinical patients’ characteristics and treatment records have emerged at an increasingly rapid pace^1^,^2^. However, such data are often very heterogeneous, high dimensional, noisy and poorly interpretable in the context of their direct usage in a clinical environment such as precise diagnostic and disease outcome prediction^2^. In addition, the data quality is often limited by small sample size, imperfect technology, difference in clinical trials, diversity of patient cohorts and health care system model. Big data analytics is the process of examining large data sets containing heterogeneous patient sub-populations and a wide variety of data types (ordinary, nominal, ordered, time series, continuous covariates). Big data analytics aims to uncover hidden patterns, unknown correlations, complex trends, imbalanced datasets, customer/clinical center preferences, geo-economic and social status, as well as other useful features^3^.

However, translation of such “averaging” characteristics (covariates) into precise disease patterns, concrete disease pathways and personalized disease prediction models is a great challenge, especially for complex genetic diseases with diverse survival risks such as cancers^4^,^5^. In our opinion, big analytics strategy for the precise personalized diagnosis and prognosis of complex genetic disease and cancer patients currently lacks in the literature. Clinically rational and feasible strategies are currently under-developed or without clear perspectives to immediate practical applications.

To develop the field of personalized medicine, it is essential to have an accurate estimation, robust and reliable prediction of an individual’s risk to disease development via a computerized data-driven approach (e.g. clinical, genomics, transcriptomes etc.). Such risk predictions can help to guide decisions to select the most effective steps for optimal disease diagnostics, monitoring and treatment.

In our previous study, we proposed the following three-step strategy of personalized cancer patient’s prognosis^6^. In the first step, the method performs sensitive, specific and robust feature selection procedure to select the most discriminative and reproducible variables (genes, clinical characteristics, etc.) that could best stratify the training set patients into two survival significant sub-groups. In this step, the variables (predictors) are converted to binary variables via maximizing the prognostic information and robustness of the selected variables^7^. In the second step, the method combines the binary variables into a multi-variable classifier of differential diagnosis, prognosis or prediction of treatment outcome^8^. In the third step, the method applies the multivariate weighted voting prognosis model to an independent patient/sample or independent dataset which were not used in the original training and attempt to classify a new patient into pre-defined classes of diagnosis, prognosis or outcome therapy risks^6^. Our method is termed Prognostic Signature Vector Matching (PSVM)^6^.

Several other classification methods exist in the literature and it is often noted that a method that demonstrates the best performance for a given patient cohort might not perform as well as another method in another cohort. The low diversity of progostic methods likely contributes some level of bias in computational prediction, especially in highly complex and heterogenous biological systems such as cancers. We propose that diversity among members of a collective predictor with a recurring classification pattern from several perspectives allows a better representation of the disease diversity than each method alone. Specifically, the implementation of a combination of different data mining and prognostic methods could be an effecient strategy for personalized and precise cancer medicine^9^,^10^,^11^,^12^.

In this study we addressed the question: how can computer prognostic models be constructed, validated and connected so that collectively they act more intelligently than any of the models? In this context, “collective intelligence” of the combined predictive system could increase the accuracy and robustness of the model due to the pooling of more predictors, and also by the increasing the quality of their interactions in the collective predictive system.

To obtain more precise and reproducible prognostic prediction for a given patient, we proposed the following updated strategy: (1) compare the PSVM with classical machine learning methods, (2) search for several classifiers, (3) and develop a multi-test voting system, which combines the best classifiers for getting more reliable and accurate personalized disease outcome prediction. Our strategy can robustly stratify HGSC patients into three survival-significant risk subgroups with distinct survival time-to-event patterns. We provided Python codes, which performed the classification analyses presented in this study.

## Results

### Signature and datasets

In our previous studies, we studied patients diagnosed with high-grade serous ovarian carcinoma (HGSC) from TCGA and identified a 36-gene combined mRNA prognostic signature which collectively stratified patients into three survival-significant risk subgroups with distinct survival time-to-event patterns^8^. These genes were biologically associated with several hallmarks of cancer, mainly cell proliferation and epithelial-to-mesenchymal transition (EMT) and correlated with chemotherapy response.

In a subsequent follow up study, we studied the effect of patients’ age on overall prognosis and due to its survival significance, we added this clinical risk factor into the original 36-gene combined mRNA prognostic signature to form a 37-variable hybrid mRNA/clinical data prognostic signature^6^.

Briefly, each of the 37 variables included in the prognostic signature was observed to be statistically significant when classifying patients into two risk subgroups via the one-dimensional data-driven grouping (1D-DDg) method (see Methods). The key parameters of the 1D-DDg prognostic models are shown in Table 1.

Subsequently, the 37 prognostic variables were combined into an integrated multivariate classifier by taking into account the contributions of all the 37 variables. This method was titled as the statistically weighted voting grouping (SWVg) method^8^. Within its implementation, the relative importance and contribution of each of the 37 variables were quantified by weights, which in our earlier studies, have been defined as the negative logarithm of the Wald’s P-values^8^. After that, each patient is assigned a prognostic score termed the weighted average risk (AWR). The patients could be classified into different risk subgroups based on the values of the AWR. The procedures were implemented in a training cohort comprising 349 TCGA HGSC patients^6^,^8^.

**Figure 1:**
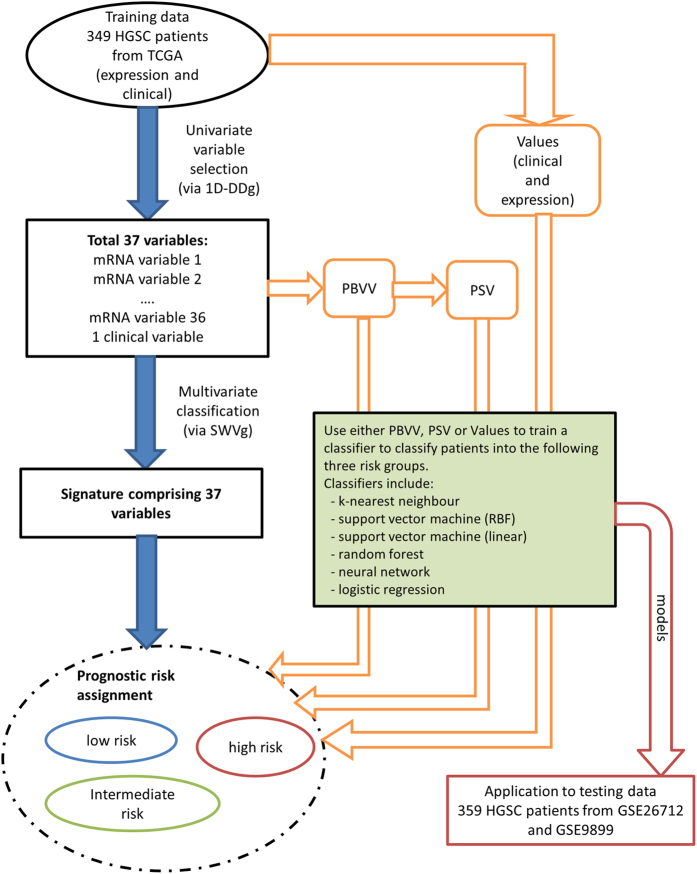
Flow chart of analyses performed in this study. HGSC patients from TCGA were used as the training data (comprising expression and clinical information). Univariate variable selection method (1D-DDg) was used to identify 37 variables which could independently stratify patients into low or high-risk. For each patient in the training cohort, the overall risk group (low, intermediate or high-risk) was summarized and assigned based on the SWVg method. Each patient can be represented by either its expression vector, PBVV or PSV. Each of the vector types was used as the variable vectors in machine learning algorithms such as k-nearest neighbour, support vector machine, random forest, neural network or logistic regression. Each of the models was assessed via 10-fold cross validation. The model was applied to an independent testing dataset comprising of 359 HGSC patients.

After that, we proposed a computational strategy to perform pairwise patients matching to identify the quantitatively most similar reference patient for each patient in the testing set. Our results proposed that Euclidean distance measure of the prognostic signature vector (PSV) is an appropriate method of assessing the similarity between any patient pair^6^. A brief schema is outlined in Fig. 1.

### Prognostic variable weights in the training dataset

In several parts of our computational procedures of personalized prognosis methods, weights were used to quantify the relative importance of variables within the multivariate classifier.

In the training dataset, weight values were initially used when combining the information from the 37 independent univariate 1D-DDg classifiers into an integrated multivariate classifier of patients’ prognostic risk. In our previous works, we have chosen to use the negative logarithm of the Wald’s test p-values to quantify the relative contribution of each variable over the other in the eventual calculation of the AWR prognostic score^6^,^8^.

However, the choice of weights can be often debatable. Here, we repeated the analyses using other weights. In the univariate classification via 1D-DDg, the two parameters that could be useful include the Wald’s test p-value (P) and hazard ratio (HR) which measures the statistical significance of the stratification and the effect size respectively. In this work, additionally, we studied the use of weights when either P or HR, or when combination of P and HR were used (Table 2). For weight A, all the variables were assigned equal weights and mathematically equally important. For weight B, the variables were assigned weights based on the inverse of its P. For weight C, variables were assigned weights based on negative logarithm of P. For weight D, the HRs was used. For weights E and F, the negative logarithm of P and HR were combined via multiplicative or summation. For each weight type (i.e. A, B, C, D, E, or F), the weights were normalized to a sum of 1 across all variables to ensure a comparable multiplicative or additive effect when different quantity measures were used in combination (Supplementary Table S1).

**Figure 2:**
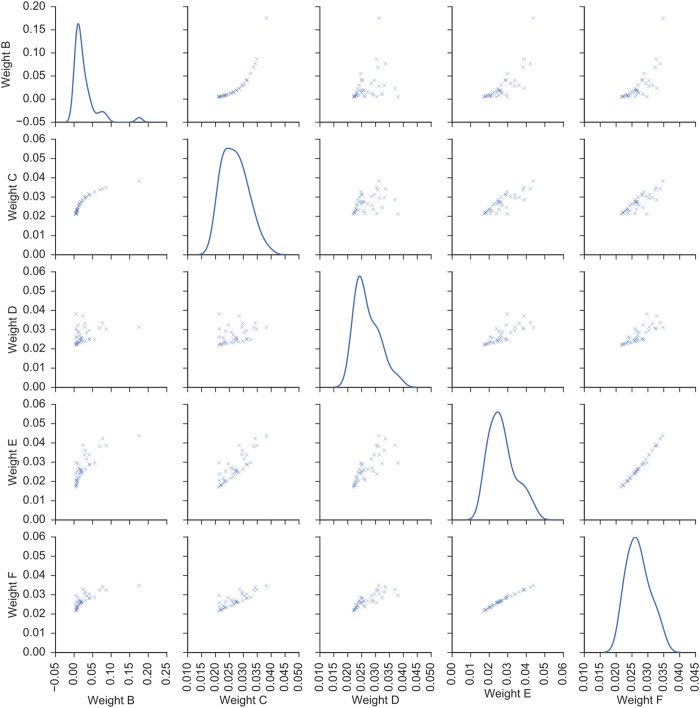
Correlation (non-diagonal plots) and distribution plots (diagonal plots) for the different weights across 37 variables. Weight (B) inverse P; Weight (C) negative log P; Weight (D) hazard ratio; Weight (E) negative log P X HR; Weight (F) negative log P + HR. The p-values were assessed via the Wald test and the hazard ratios of the individual variables were assessed via the Cox Proportional Hazards model.

The correlation plots for the different pairs of weight types across all the variables are shown in Fig. 2. The weight that uses inverse P has the largest ratio of the biggest weight to the smallest weight (ratio = 34.7, Supplementary Table S1). This suggests that in the combinatory signature, most of the contribution would be due to the top few variables. To alleviate the issues of the potentially large dynamic range of the significance of the Wald’s test p-values, the negative log representation of this quantity will reduce the dynamic range, making it more feasible as variable weights.

**Figure 3:**
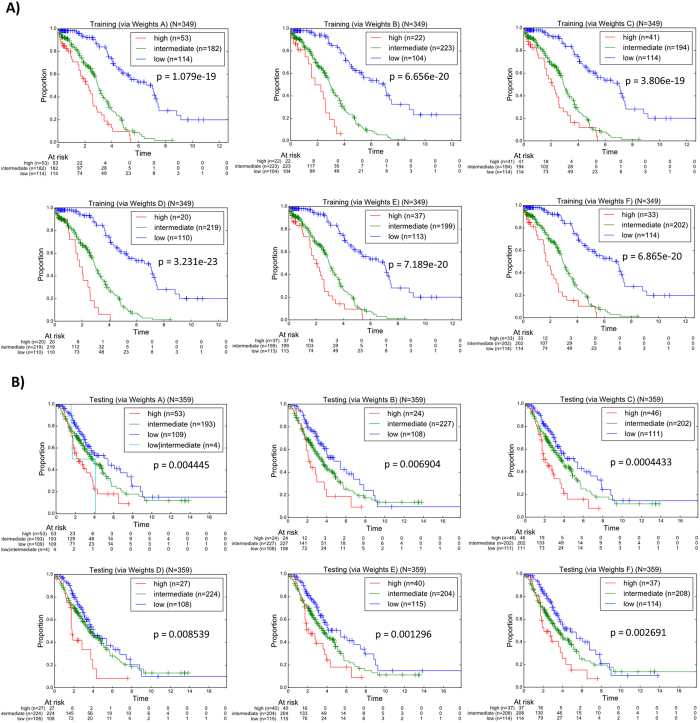
(**A**) Training classification of the TCGA via SWVg method with different weight parameters. (**B**) Testing classification of the GSE26712 and GSE9899 patient cohorts via matching to the nearest reference patient from the training cohort. Weight (**A**) constant weight; Weight (**B**) inverse P; Weight (**C**) negative log P; Weight (**D**) hazard ratio; Weight (**E**) negative log P X HR; Weight (**F**) negative log P + HR. The p-values were assessed via the Wald test and the hazard ratios of the individual variables were assessed via the Cox Proportional Hazards model.

The overall classification plots of the training cohort via the SWVg method from the use of the different weight parameters are shown in Fig. 3A.

These results revealed that in the training cohort, summarization of the risk scores from the various and independent 1D-DDg classifier via a weighted voting approach can yield strong classification of the distinct prognostic risk subgroups. Apart from minor statistical and visual differences, strong prognostic stratifications were observed when any of the choices of weights were used (p ≤ 5.0E-19). Of note, if additional criteria are required, such as minimization of the size of the intermediate-risk subgroup and maximization of size the high-risk subgroup, then Weight A (equal weights) and Weight C (-log P) might be preferred for our dataset (Fig. 3A).

### Prognostic variable weights in the test dataset

To apply the prognostic classifier information from the training to the testing set, we have proposed the following procedures that are outlined as a schema in Fig. 1. The cutoff values and designs from each of the univariate classifier were applied to the testing set to assign patients to low or high risk. Thereafter, each patient in both the training and testing cohort could be represented by a prognostic binary variable vector (PBVV), which is then converted to a prognostic signature vector (PSV)^6^. The generation of PSV from PBVV is proposed to consider the ranks of the individual variables based on their respective and relative importance. For each query patient in the testing cohort, the PSV of that query patient could be compared to the PSVs of all reference patients in the training cohort. Upon finding a quantitatively most similar reference patient, the query patient is predicted to exhibit the overall prognostic risk of that most similar reference patient^6^.

In our PSVM model of personalized prognosis, prior to converting the prognostic binary variable vector to prognostic signature vector, we have also proposed that the variables are ranked based on their respective variable weights that were defined as the negative logarithmized Wald’s p-value. In this study, we investigated the overall effect of survival prediction in the testing cohort when other weight measures were used (Table 2). The K-M plot and Wald’s p-value results are shown in Fig. 3B. These results indicated that in the test cohort, the use of negative log P (Weights C) as weights provide the best stratification (p = 0.00044), when compared to the other weight choices (Fig. 3B). Importantly, the proportions of the numbers of patients of the high-, intermediate- and low- risk subgroups in the test data (0.117, 0.556, 0.327) are very similar the ones in training data (0.128, 0.563, 0.309).

### Application of alternative classification approaches involving classical machine learning techniques

From the training dataset, we have defined an overall prognostic risk for each patient (low-, intermediate- or high- risk), as well as representing each patient as a prognostic binary variable vector (PBVV) via the 1D-DDg algorithm. The PBVV could be converted to PSVs as described in our previous work^6^,^8^.

Next, we studied the accuracies of classical machine learning algorithms to train and perform class prediction based on either PBVVs, PSVs or the original expression/clinical data vector (Fig. 1).

In this comparative analysis, our PSVM method uses un-scaled PSV variables (as it was designed) whereas the machine learning techniques uses scaled PSV variables (standard scaling to zero mean and unit variance). This is to ensure that each of the feature variables was accorded equal contribution to the classifier during training.

**Figure 4:**
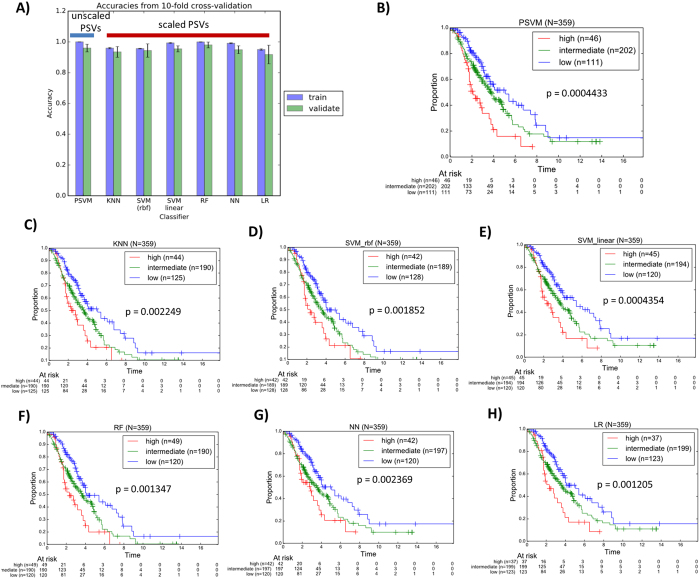
Training, validation and testing of the classifier. (**A**) Mean accuracies of the ten-fold cross validation of our method (PSVM) as well as for each machine learning algorithm when applied to TCGA training cohort comprising 349 HGSC patients. The vertical bars represent the standard deviation of the accuracy values. The PSVM method is based on un-scaled PSV variables whereas the classical machine learning techniques were based on scaled PSV variables. Classification curves of the testing dataset from (**B**) our PSVM method, in comparison with other classical machine learning algorithms including (**C**) k-nearest neighbor, (**D**) support vector machine (RBF kernel), (**E**) support vector machine (linear kernel), (**F**) random forest, (**G**) neural network and (**H**) logistic regression. The p-values were assessed via log-rank test. Abbreviations: OURS – our method; KNN – k-nearest neighbor; SVM-RBF – support vector machine with radial basis function kernel; SVM-linear – support vector machine with linear kernel; RF – random forest; NN – neural network; LR – logistic regression.

We performed a ten-fold cross validation analysis where the training cohort was split into ten subsets. For each of the ten subsets, it was used as the validation set whereas the remaining nine subsets were used as the training set. Our results revealed that all the methods exhibited relatively high accuracy in the training as well as ten-fold cross validation in the training dataset (Fig. 4A, Supplementary Table S2). The RF and PSVM methods showed the most significant accuracy. We repeated the 10-fold cross validation analyses several times, via shuffling the data before splitting the data into training and validation set. Our results across different ten-fold cross validation analyses revealed very similar accuracy values (Supplementary Table S2).

The classifiers were then applied to the independent testing dataset and the Kaplan-Meier survival curves of each of the algorithms are shown in Fig. 4B–H (Supplementary Table S3). The significance of the stratification in the test set, defined by log-rank test p-value, was consistent with that of the accuracy in the training cohort. The risk class predictions in the independent test cohort were relatively consistent and showed reproducible overall survival prognostic patterns.

### Voting of the diverse survival classification models and their output correlations

**Figure 5:**
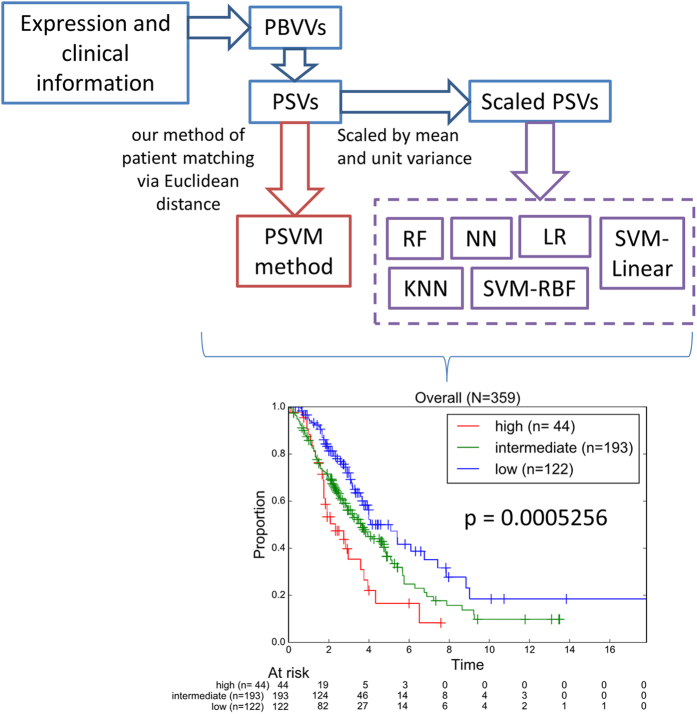
Kaplan-Meier plots of patients’ classification from the independent test cohort. The grouping information was obtained from the combination of grouping information from our PSVM method (derived from un-scaled PSVs) and the five machine learning techniques (KNN, SVM-RBF, SVM-linear, RF, NN, LR, trained from scaled PSVs).

Finally, we performed a voting procedure based on the ensemble of 7 independent classification methods, to generate an overall prognostic risk classification of the patients in the testing set (Fig. 5). The overall risk prediction for each patient was defined by the most predicted class via the 7 independent classification methods. Our results revealed strong significant stratification of the test patients into low, intermediate and high risks (p = 0.0005256). Using Supplementary Table S3 data, we demonstrated that the results of the classifier’s outputs are strongly correlated to each other (Table 3). Correlation analysis demonstrated that linear support vector machine (SVM_linear) and PSVM may be considered as the most representative classifiers, contributing mostly onto the output of the ensemble (multi-test) voting procedure (Table 3).

In view of the inherent biasness of each of the classification algorithms and the high complexity of the underlying datasets, the accuracy and performance of the risk class prediction might widely vary. In these circumstances, our analysis demonstrated that a voting of the output categorical values for a given patient across distinct prognostic/classification methods could lead to more robust, accurate, reproducible and cost efficient prognostic/classification strategy for precise medicine.

## Discussion

In this work, we extend on our previously developed methods of personalized prognosis. Our primary single variable classifier is the one-dimensional data-driven grouping (1D-DDg) method, which stratifies a cohort of patients into two survival significant subgroups: low or high-risk. The 1D-DDg method itself is optimized, i.e. for a single variable permissible domain, it identifies a cut-off value that splits the domain into low and high sub-domains and, subsequently, the patient cohort into two distinct subgroups with the most statistically significant Wald’s test p-value.

During the process of identifying prognostic significant variables, the 1D-DDg method should be considered a complementary approach to methods which utilizes the whole permission domain (range) of the continuous expression values^5^. In many analyses such as regression or correlation, it would be beneficial to analyze the continuous variable values with respect to the dependent variable. However for prognosis analysis where dependent variable, i.e. prognostic outcomes (low or high risk) is normally not known *a priori*, the 1D-DDg method could be a reasonable first step. The 1D-DDg 1) identifies an optimal cutoff value of the predictor and 2) to assign patients to low or high risk depending on whether the patients’ predictor expression value lie on the leftwards or rightwards of the optimal cutoff value and the pattern of association. In particular, dichotomization based on a pre-specified cut-point is common in many areas of clinical research or clinical diagnosis, where patients are routinely diagnosed based on the category of his/her measurement^13^. It is also expected that the model derived from 1D-DDg would yield easily understandable and useful cut-points, potentially with clinical applications.

In the first part of our study, we investigated the effects of different weights on SWVg prognostic classification algorithm performance (as quantitatively assessed by the multivariate log-rank test). Our results revealed that the choice of weights when combining several independent univariate variables into a multivariate classifier is relatively important. In our study, the use of negative logarithmized p-values appears to outperform other weights involving hazard ratios (Fig. 3). The choice of weights should be considered an essential task in any combination/voting procedures.

In the second part of our study, we extended our analysis to the use of classical machine learning techniques to train a classifier that delineates between risk classes stratified via our 1D-DDg and SWVg prognostic classification algorithm. We studied the use of classical machine learning techniques such as support vector machine, k-nearest neighbor, logistic regression, random forest and neural networks. For each classification method, we made a preliminary attempt to optimize the parameters via grid searching of each of the parameters, and optimization of the cross validation accuracy. It is commonly appreciated that variables should be scaled before application of machine learning algorithms. In such cases, we observed that when the PSVs are scaled, the training and cross validation accuracies across the classical machine learning techniques such as KNN, SVM, RF, NN and LR were relatively high. PSVM method was designed and proven to work with the PSVs directly without variable scaling^6^. For most cases, the cross validation accuracies are generally high enough without the need for extensive and finer tuning (Fig. 4A).

Also, our results showed that the PSVM algorithm that we have developed in our previous work could marginally outperform most other classical machine learning algorithms including k-nearest neighbor, support vector machine, logistic regression and neural network (Fig. 4B–H). Nevertheless, we propose that an ensemble approach that considers the classification results from all the independent classifiers (ours and that from the classical machine learning algorithms) could be a way to unify and address concerns that results from a single classifier is likely to be biased, non-robust, and produce irreproducible results in the test set. Our results revealed that if such schema were used, the overall classification would provide relatively significant stratification of the patients from the independent test cohort (Fig. 5). We suggest that such an ensemble (multi-test) approach is likely to lead to more robust and reproducible results. Such approach could be more feasible, rather than efforts to micro optimize a classifier of choice which might not work well in another dataset or patient cohort.

In this current work, we have focused our analyses on a prognostic signature that we have previously identified in HGSC tumors and subsequently determined to be associated with regulation of cell-cycle control and epithelial-to-mesenchymal transition which are two of the cancer hallmarks previously elucidated by Hanahan and Weinberg^14^. Our progress in the field of personalized prognosis in HGSC is aligned with current trends in biomarker discovery using cancer hallmarks or a combinatory set of cancer hallmark signatures to improve prediction accuracy^5^,^15^. Our future efforts will be focused on incorporating various conventional cancer hallmark signatures with the use of different classification algorithms to predict patients’ overall survival risk in a more robust and less biased manner.

Importantly, our HGSC prognostic variables of interest include both mRNAs and clinical data. In view of emerging evidence that long non-coding RNAs, miRNAs, epigenetic signals or mutations could play critical roles in prognosis and potentially personalized prognosis^8^,^16^,^17^,^18^, future research efforts should also be focused on incorporating these into a multi-type, multi-variable and multi-classifier signatures.

To surmise, while we have previously reported a formal description of PSVM method of personalized prognosis, in this work, we additionally studied the effect of using various prognostic variable weights when defining the PSVM. We further compared our method, with other variations of the method, as well as with other prediction methods (classifiers) using classical machine learning techniques. The search for “an ideal” patient classification algorithm based on genomic or clinical features that works across different datasets and diseases is often complicated by the fact different classification algorithm works well in specific situations and are sensitive to sample size and the data input. Voting strategy of the outputs from the different and independent classifiers could result in more robust and reproducible patients’ risk classification for the individual patients. The use of such multi-test diagnostic or prognostic collective intelligence system approach, rather than the search for the ideal classification/prediction method, might help to address the current clinical needs for robust and reproducible classification. The fields of personalized prognosis and precise prediction in medicine are rapidly growing ones. We suggest that our collective intelligence computational method could improve the analytics strategy for translation of automatic prognostic systems into practical clinical oncology needs. We have provided Python codes, which performed the classification analyses presented in this study.

## Methods

### Dataset

We studied publicly available gene expression and clinical datasets of HGSC patients from three patient cohorts: TCGA^19^, GSE9899^20^ and GSE26712^21^. For each patient, gene expression was profiled via Affymetrix U133A microarray and clinical characteristics of each patient including but not limited to overall survival time, vital status, age at initial diagnosis and therapy outcome success were collected.

### Assigning class labels to training set - Univariate classifier

We used a published 1D-DDg algorithm as the single variable classifier of patients’ overall survival times and events^7^. The 1D-DDg algorithm is applied for a single variable across the patients. Briefly, the patients are ranked based on the value of the variable (predictor). For each variable, a 1D-DDg permissible domain is defined. Such domain is the set of possible variable values of an attribute (e.g. mRNA expression or clinical variable such as age). Subsequently, a cut-off value of the variable stratifies the patient cohort into two subgroups with the most statistically significant distinct survival patterns. The overall survival times and events from the two stratified patient subgroups can be visualized via Kaplan-Meier survival plots, and the statistical significance between the low-risk and high-risk subgroups can be quantified by 1D-DDg method via p-value from the Wald’s test^7^.

The implementation of 1D-DDg algorithm for the i^th^ variable across M patients can be described mathematically as follows:


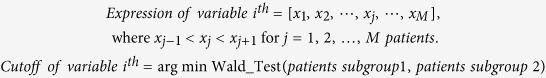


Further details could be found in our previous work^7^.

### Assigning class labels to training set - Multivariate classifier

While 1D-DDg is a univariate classifier of overall survival based on a gene or clinical variable, several of it could be combined into a multivariate classifier. We proposed the use of SWVg method to combine several independent 1D-DDg classifiers^8^.

The selection of the variables that make up the overall multivariate classifier is beyond the scope of our current discussion, and the interested reader is encouraged to refer to our previous reports^6^,^8^. However briefly, a subset of variables could be selected from the unbiased screening of all available variables (e.g. gene variables from microarray expression profiling or clinical characteristics of patients) based on either their prognostic significance or biological significance. The top statistically discriminative variables, together with prior biological knowledge about the variables, could assist in the selection of variables that in combination could stratify patients into multiple subgroups of distinct risk patterns.

In multivariate classification, each patient is assigned a prognostic risk score called average weighted risk (AWR) that is the average of all the risk score assigned by each of the selected and independent 1D-DDg classifiers. Essentially, the prognostic risk score for any given is voted by several independent raters, i.e. several independent 1D-DDg classifier. Therefore the method is termed voting grouping method. When weights are assigned to each of the 1D-DDg univariate classifier during the averaging procedure, the method then becomes statistically weighted voting grouping (SWVg) method^8^.

For survival classification problems with the eventual goal of stratifying a patient cohort into statistically significant and distinct subgroups, an intuitive choice for the selection of weights would be the negative logarithmized log-rank p-values of the stratification performance for that univariate classifier. However depending on the application, other possible choices may include hazard ratios or magnitude of fold-change^6^.

After each patient has been assigned a prognostic risk score via the independent 1D-DDg classifiers, the patients are ranked via the prognostic risk score. Subsequently, thresholds of the prognostic risk scores are identified to identify subgroups of patient stratification with survival significance^8^.

### Training of a classifier

The methods of 1D-DDg and SWVg were initially used to assign class labels to each of the reference patient samples of the training cohort, depending on whether they are categorized as low, intermediate or high-risk. If 1D-DDg and SWVg were used as the risk assignment methods, the patient cohort would be reliably stratified into subgroups with distinct Kaplan-Meier survival curves.

After the assignment of the class labels to each sample, we then used classical machine learning techniques to train a classifier that delineates these subgroups in the training and validation dataset with high accuracies. Such methods typically involve classical machine learning approaches such as support vector machine (SVM), neural networks (NN), k-nearest neighbor (KNN), random forest (RF), and logistic regression (LR), etc. We evaluated each of these methods and compared the statistics from the use of each of these machine learning techniques.

### Cross-validation analysis

We performed 10-fold cross validation analysis on the training set. The training set was splitted into ten folds with the maintenance of the class ratios in each fold, i.e. the proportion of class 1 (low-risk), class 2 (intermediate-risk) and class 3 (high-risk) in all the folds were maintained at 55.6%, 32.7% and 11.7% respectively in each of the ten folds. For each of the 10 subsets, it was used as the validation set whereas the classifier was trained on the remaining 9 subsets.

We repeated the 10-fold cross validation analyses several times, via shuffling the data before splitting the data into training and validation set.

The multi-class classifications for the various machine learning techniques were performed via the basis of one-versus-rest for all the classes independently. In this strategy, one classifier was fitted for each class (e.g. class 1) against all the other classes (e.g. class 2 and class 3).

The metric classification accuracy was used as the evaluation statistic.

### Testing of a classifier

The classifier is applied to an independent testing dataset which comprise 359 HGSC patients from GSE9899 and GSE26712.

During class prediction of each of the samples of the independent testing dataset, the predicted class is determined by the consensus from the ten classifiers of ten folds cross validation obtained during the classifier training.

We evaluated the test classification’s overall survival pattern via Kaplan-Meier curves and assessed the survival significance of the differential risk groups via log-rank test^22^,^23^.

### Implementation

The computational analyses were carried out using Python programming language and the following open source libraries: pandas (data manipulation), scikit-learn (machine learning), lifelines (survival analysis) and matplotlib/seaborn (visualization).

### Weights are important

Wald test p-values (P) are commonly used as a normalization factor to weigh the significance and relative importance of different variables. Here, we studied the effect of using hazard ratio (HR) as weight. We also studied the combinatory use of both hazard ratio and the Wald’s test p-value as weights. In summary, the following weights were assessed:


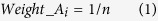



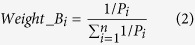



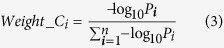



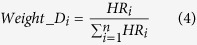



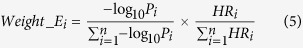



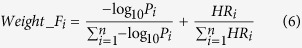


where *P*_*i*_ and *HR*_*i*_ denotes the Wald’s test p-value and the hazard ratio respectively for the *i* = 1, 2, 3, ···, *n*^*th*^ variable.

### Variable Scaling

Machine learning techniques typically necessitates the scaling of features. The values of PSV’s variable i^th^ are scaled to zero mean and unit variance by the following:


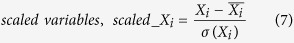


This procedure was implemented in scikit-learn preprocessing module.

### Software

We have provided Python codes, which performed the classification analyses presented in this study. The codes, readme file and examples are presented in Supplementary Codes and Examples. This folder contains the codes to perform classifier training of specified variables (in this case, prognostic signature variables) to samples that have been labelled as “low”, “intermediate”, or “high”. The training and 10-fold cross validation of the classifiers was performed on the reference/training dataset. The trained classifier was applied to an independent test set for risk class prediction.

## Additional Information

**How to cite this article**: Ow, G. S. and Kuznetsov, V. A. Big genomics and clinical data analytics strategies for precision cancer prognosis. *Sci. Rep.* **6**, 36493; doi: 10.1038/srep36493 (2016).

**Publisher’s note:** Springer Nature remains neutral with regard to jurisdictional claims in published maps and institutional affiliations.

## Supplementary Material

Supplementary Information

Supplementary Information

Supplementary Information

Supplementary Information

## Figures and Tables

**Table 1:** Parameters of one-dimensional data-driven grouping (1D-DDg) method for selected mRNA and clinical variables when applied to the training set comprising 349 TCGA patients diagnosed with high-grade serous ovarian cancer (HGSC).

#	Variable	Desc	P-value (Wald)	Cutoff	Design	Variable behaviour	Hazard ratio	#Low risk	#High risk
1	205382_s_at	*CFD*	0.0003721	8.026	2	onco-like	2.024	302	47
2	202246_s_at	*CDK4*	0.0007521	9.05	1	supp-like	1.964	310	39
3	204451_at	*FZD1*	0.0008483	6.66	2	onco-like	2.177	55	294
4	201947_s_at	*CCT2*	0.0009464	10.805	2	onco-like	2.003	312	37
5	205959_at	*MMP13*	0.0011793	6.618	2	onco-like	1.606	146	203
6	201954_at	*ARPC1B*	0.0015679	9.175	2	onco-like	1.617	245	104
7	age	age	0.0015858	67	2	onco-like	1.641	258	91
8	201615_x_at	*CALD1*	0.0016123	7.976	2	onco-like	1.917	63	286
9	204464_s_at	*EDNRA*	0.0019353	8.037	2	onco-like	1.845	311	38
10	208944_at	*TGFBR2*	0.0021252	8.281	2	onco-like	1.565	161	188
11	203968_s_at	*CDC6*	0.0021869	6.108	1	supp-like	2.153	325	24
12	209026_x_at	*TUBB*	0.0022915	10.051	1	supp-like	2.031	322	27
13	201774_s_at	*NCAPD2*	0.0027595	8.598	2	onco-like	2.401	324	25
14	212239_at	*PIK3R1*	0.0028506	7.992	2	onco-like	1.535	195	154
15	203131_at	*PDGFRA*	0.0034062	8.876	2	onco-like	1.678	283	66
16	212063_at	*CD44*	0.0034184	8.845	1	supp-like	1.574	125	224
17	212782_x_at	*POLR2J*	0.0034369	10.432	2	onco-like	1.615	278	71
18	214144_at	*POLR2D*	0.0034777	7.255	1	supp-like	1.657	90	259
19	219588_s_at	*NCAPG2*	0.0042218	8.342	1	supp-like	1.507	185	164
20	209960_at	*HGF*	0.0043267	6.522	2	onco-like	1.764	310	39
21	212294_at	*GNG12*	0.0043607	9.312	2	onco-like	1.614	283	66
22	207822_at	*FGFR1*	0.0050334	6.083	2	onco-like	1.865	62	287
23	204441_s_at	*POLA2*	0.0052135	7.167	1	supp-like	1.511	150	199
24	216598_s_at	*CCL2*	0.0062289	6.453	2	onco-like	2.101	29	320
25	202107_s_at	*MCM2*	0.0069779	8.671	1	supp-like	1.484	232	117
26	202202_s_at	*LAMA4*	0.0076547	8.863	2	onco-like	1.893	318	31
27	215076_s_at	*COL3A1*	0.0080398	11.322	2	onco-like	1.504	250	99
28	210845_s_at	*PLAUR*	0.0082985	7.446	2	onco-like	1.555	93	256
29	201697_s_at	*DNMT1*	0.0088655	9.143	1	supp-like	1.47	148	201
30	202877_s_at	*CD93*	0.0090467	6.844	2	onco-like	1.675	60	289
31	203323_at	*CAV2*	0.0103597	7.806	2	onco-like	1.442	204	145
32	221559_s_at	*MIS12*	0.0109485	7.822	1	supp-like	1.471	227	122
33	208778_s_at	*TCP1*	0.0110947	9.455	2	onco-like	1.452	142	207
34	201091_s_at	*CBX3*	0.0120077	9.928	1	supp-like	1.701	65	284
35	205393_s_at	*CHEK1*	0.0120697	6.128	1	supp-like	1.975	328	21
36	200931_s_at	*VCL*	0.0125254	9.888	2	onco-like	2.471	22	327
37	212949_at	*NCAPH*	0.0129085	7.163	2	onco-like	1.434	214	135

The statistical significance of patients’ classification into two risk groups based on an optimized expression cutoff value was measured via the Wald’s test, whereas the hazard ratio was calculated from the beta coefficient from the Cox Proportional Hazards model. Design: 1 - low expression, high-risk; 2 - low expression, low-risk. Abbreviations: onco – oncogenic; supp – suppressive.

**Table 2:** Choices of variable weight to signify the relative importance and contribution of each variable in entire multi-variable classifier.

Weight name	Description	Equation
A	Constant weight	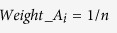 (1)
B	Inverse of P	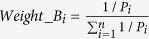 (2)
C	Negative logarithm of P	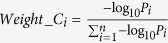 (3)
D	Hazard ratio	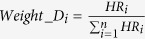 (4)
E	Negative logarithm of P X HR	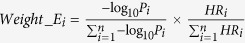 (5)
F	Negative logarithm of P + HR	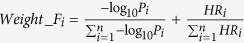 (6)

Abbreviations: P_i_ and HR_i_ denotes the Wald’s test p-value and the hazard ratio respectively for the i = 1, 2, 3, …, n^th^ variable.

**Table 3:** Gamma correlation coefficient value matrix demonstrates high consistency between PSVM and the other methods, which accurately stratify the patient of testing cohort onto low-, medium- and high- risk subgroups of the disease outcomes.

Gamma Corr	Time	Event	PSVM	KNN	SVM_rbf	SVM_linear	RF	NN	LR	Overall
Time	1.00	−0.24	0.07	0.01	0.01	0.03	0.05	−0.02	−0.01	0.029
Event		1.00	0.03	0.08	0.11	0.09	0.10	0.11	0.10	0.098
PSVM			1.00	0.95	0.95	0.98	0.93	0.92	0.90	**0.984**
KNN				1.00	0.97	0.96	0.91	0.91	0.88	0.971
SVM_rbf					1.00	0.96	0.93	0.91	0.90	0.972
SVM_linear						1.00	0.96	0.94	0.91	**0.992**
RF							1.00	0.92	0.91	0.951
NN								1.00	0.93	0.945
LR									1.00	0.920
Overall										1.000

Suppl. Table S3 data was analyzed. Gamma correlation coefficients were calculated using STATISTICA-6 software. (n = 359; P < 0.05).
