# Supplementary material for: Big genomics and clinical data analytics strategies for precision cancer prognosis
Source: Sci Rep. 2016 Nov 7;6:36493. doi: 10.1038/srep36493 (PMC5098145; doi:10.1038/srep36493)
Supplement: Supplementary Information [file srep36493-s4.zip › Supplementary codes and examplesGSO_VK/Example_Results/Survival Plots - NN_scaled.pdf]

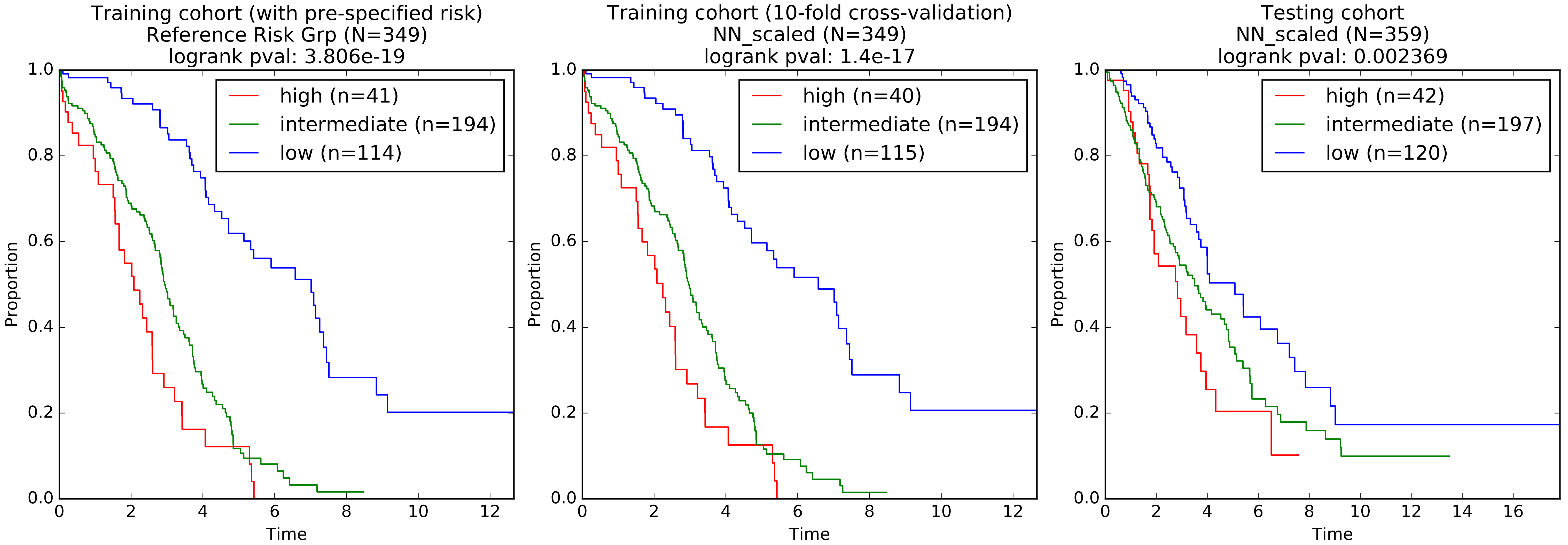

Training cohort (with pre-specified risk)
Reference Risk Grp (N=349)
logrank pval: 3.806e-19
high (n=41)
intermediate (n=194)
low (n=114)
Proportion
Time
Training cohort (10-fold cross-validation)
NN_scaled (N=349)
logrank pval: 1.4e-17
high (n=40)
intermediate (n=194)
low (n=115)
Proportion
Time
Testing cohort
NN_scaled (N=359)
logrank pval: 0.002369
high (n=42)
intermediate (n=197)
low (n=120)
Proportion
Time
