# Supplementary figures and images for: Big genomics and clinical data analytics strategies for precision cancer prognosis
Source: Sci Rep. 2016 Nov 7;6:36493. doi: 10.1038/srep36493 (PMC5098145; doi:10.1038/srep36493)

Accuracies from 10-fold cross-validation  
error bars: std. dev.

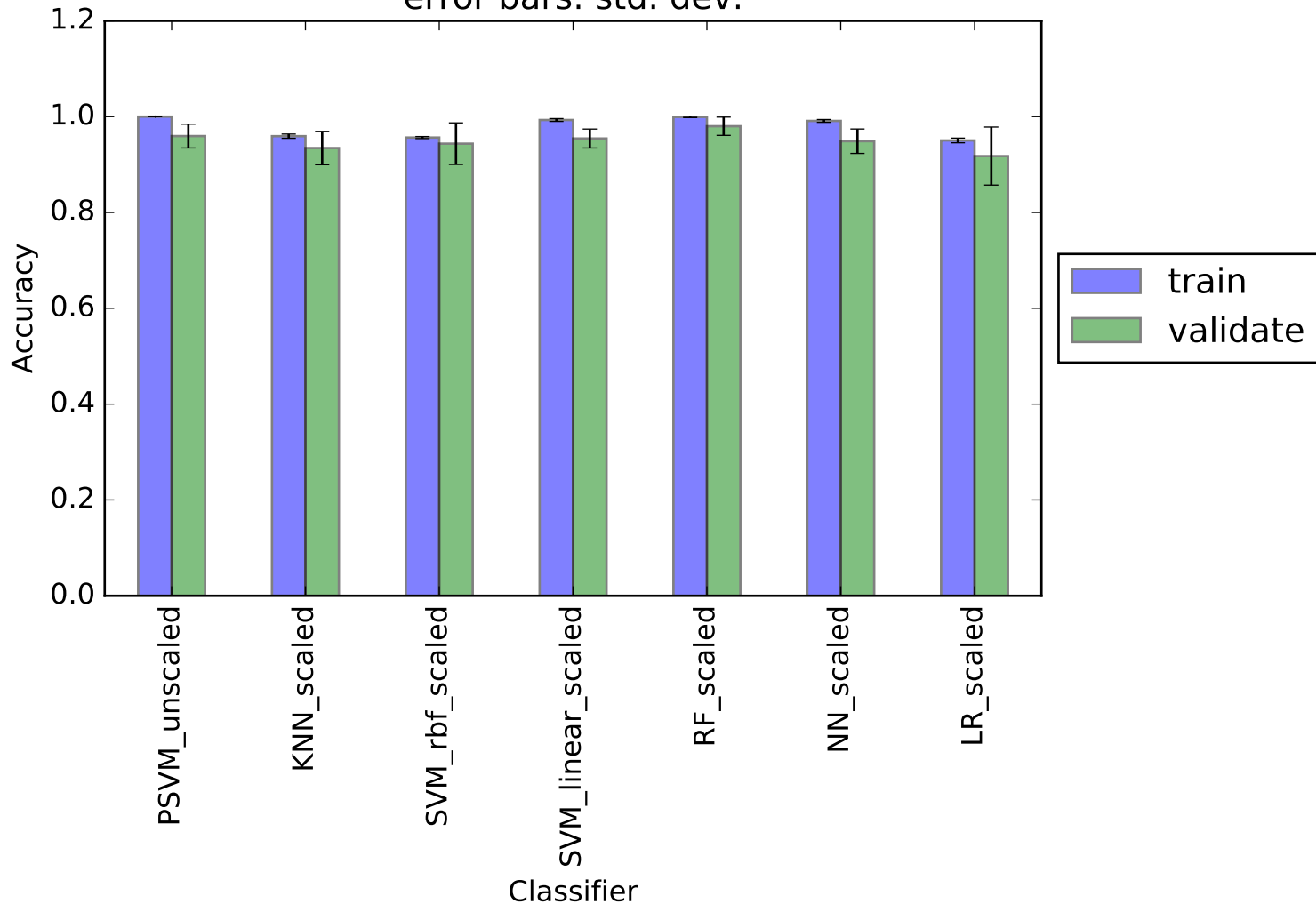

Supplement: Supplementary Information [file srep36493-s4.zip › Supplementary codes and examplesGSO_VK/Example_Results/Accuracy.pdf]

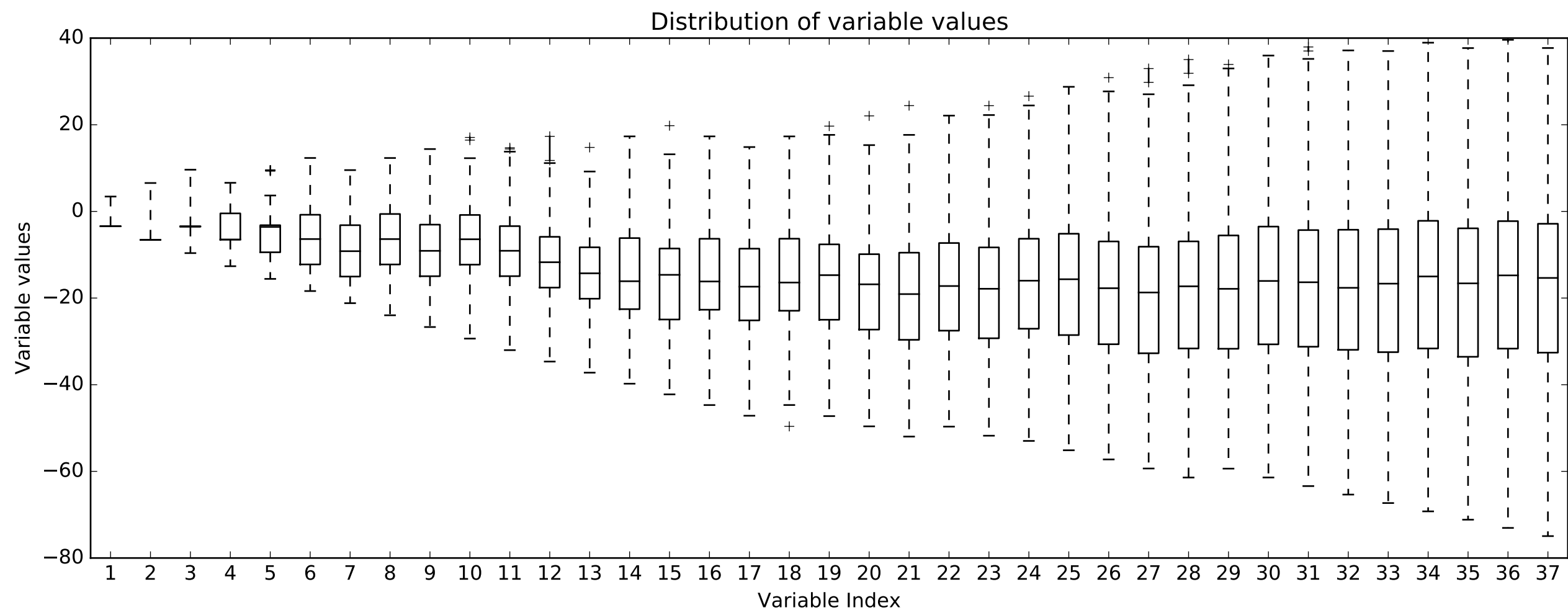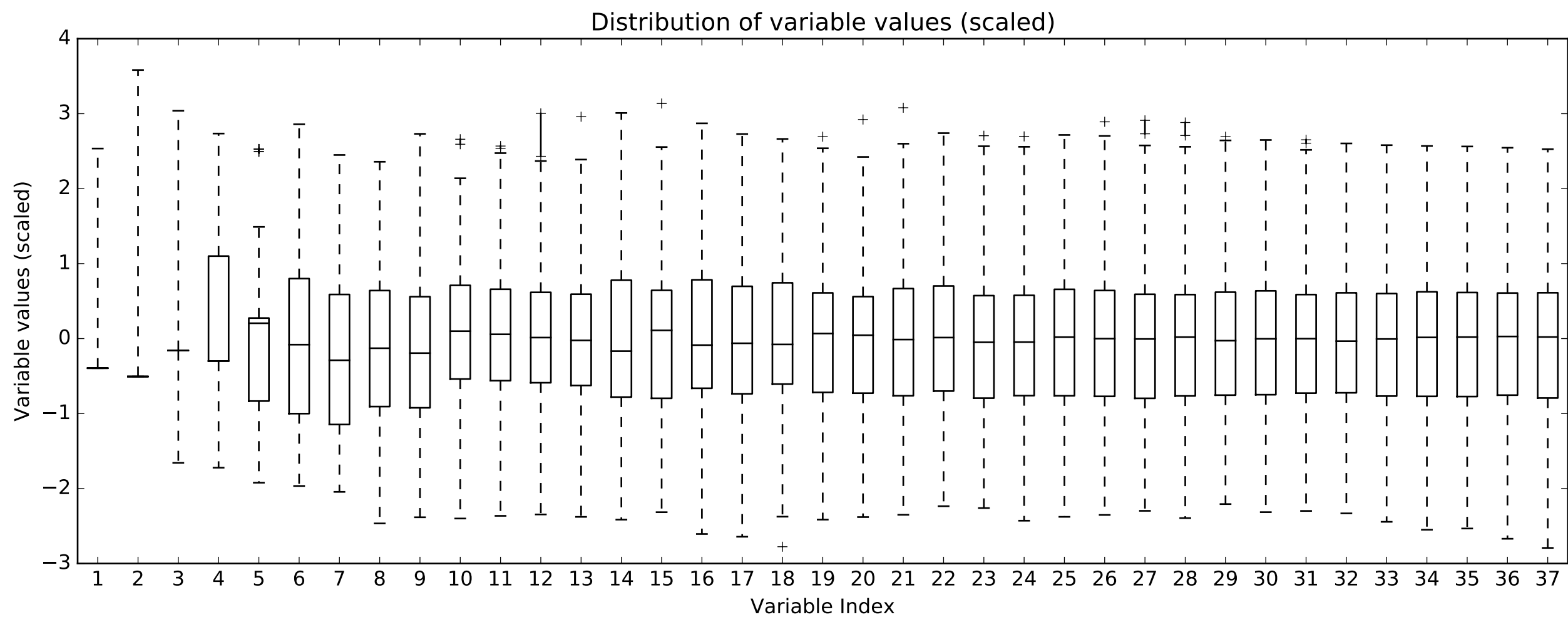

Supplement: Supplementary Information [file srep36493-s4.zip › Supplementary codes and examplesGSO_VK/Example_Results/Distribution.pdf]

Overall  
Overall (N=359)  
logrank pval: 0.0005256

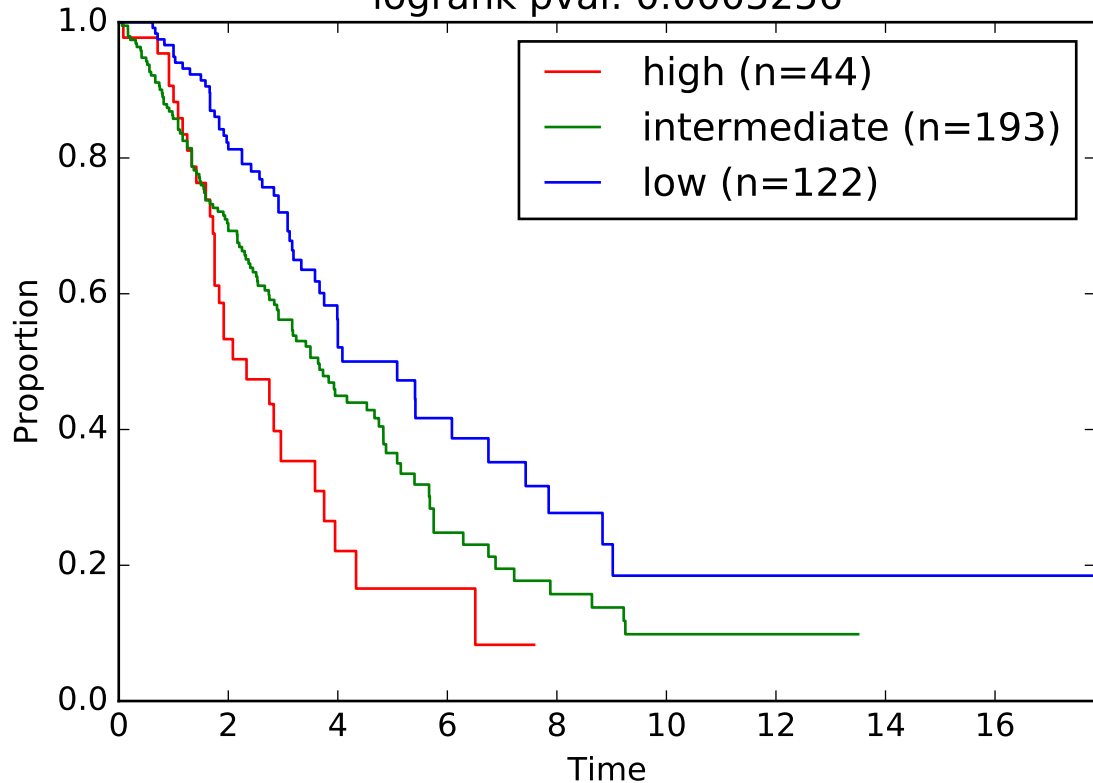

Supplement: Supplementary Information [file srep36493-s4.zip › Supplementary codes and examplesGSO_VK/Example_Results/Survival Plots - Overall.pdf]
